# The Health Status Transition and Medical Expenditure Evaluation of Elderly Population in China

**DOI:** 10.3390/ijerph18136907

**Published:** 2021-06-27

**Authors:** Lianjie Wang, Yao Tang, Farnaz Roshanmehr, Xiao Bai, Farzad Taghizadeh-Hesary, Farhad Taghizadeh-Hesary

**Affiliations:** 1 School of Public Administration, Zhejiang University of Finance and Economics, Hangzhou 310018, China; wanglianjie@zufe.cn; 2 Shibata Laboratory, School of Advanced Science and Engineering, Waseda University, Tokyo 162-8480, Japan; f.roshanmehr@kurenai.waseda.jp; 3 Kagawa Nutrition University, Saitama 350-0288, Japan; 4 School of Finance, Zhejiang University of Finance and Economics, Hangzhou 310018, China; baixiao@zufe.edu.cn; 5 Clinical Oncology Department, Shahid Beheshti University of Medical Sciences, Tehran 19857-17443, Iran; f_taghizadeh@sbmu.ac.ir; 6 Social Science Research Institute, Tokai University, Tokyo 259-1292, Japan

**Keywords:** aging population, elderly health transition, long-term care, medical expenditure

## Abstract

(1) Background: Because of the rapid expansion of the aging population in China, their health status transition and future medical expenditure have received increasing attention. This paper analyzes the health transition of the elderly and how their health transition impacts medical expenditures. At the same time, feasible policy suggestions are provided to respond to the rising medical expenditure and the demand for social care. (2) Methods: The data were obtained from the China Health and Retirement Longitudinal Study (CHARLS) from 2011 to 2015 and analyzed using the Markov model and the Two-Part model (TPM) to forecast the size of the elderly population and their medical expenditures for the period 2020–2060. (3) Results: The study indicates that: (1) for the elderly with a mild disability, the probability of their health improvement is high; in contrast, for the elderly with a moderate or severe disability, their health deterioration is almost certain; (2) the frequency of the diagnosis and treatments of the elderly is closely related to their health status and medical expenditure; alternatively, as the health status deteriorates, the intensity of the elderly individuals’ acceptance of their diagnosis and treatment increases, and so does the medical expense; (3) the population of the elderly with mild and moderate disability demonstrates an inverted “U”-shape, which reaches a peak around 2048, whereas the elderly with severe disability show linear growth, being the target group for health care; (4) with the population increase of the elderly who have severe disability, the medical expenditure increases significantly and poses a huge threat to medical service supply. Conclusions: It is necessary to provide classified and targeted health care according to the health status of the elderly. In addition, improving the level of medical insurance, establishing a mechanism for sharing medical expenditure, and adjusting the basic demographic structure are all important policy choices.

## 1. Introduction

From a global perspective, the health risks of the population aging and rising medical expenditure have an important impact on socioeconomic development, which has aroused widespread concerns. Since the beginning of the 21st century, China’s demographic structure has undergone drastic and profound changes alongside the deepening of economic transformation and social transition. The increasing proportion of the aging population is undoubtedly one of the most critical features of China’s demographic transformation. According to the statistics, China’s population of elderly people over 60 increased from 167 to 264 million from 2009 to 2020, an average annual increase of about 8.8 million. In addition, this number will exceed 400 million by 2050, which will account for more than 30% of the total population [1]. One of the changes with this aging trend is the health status transition. In the past ten years, the average annual growth rate of the elderly population aged 80 and above in China has been 4.7%, which is significantly faster than those who are 60 and above. Because the physical functions of the elderly are gradually weakened as they grow older, the population of disabled and semi-disabled elderly will grow rapidly. At the end of 2019, statistics showed that among the 254 million elderly population in China, about 120 million elderly people suffered from chronic diseases, and the disabled and semi-disabled elderly population exceeded 40 million [2]. Due to the risks of the health deterioration of the elderly, their demand for medical services is on the rise, along with medical expenditure. In this paper, the relationship between the health status and medical expenditure of the elderly is discussed from both individual and household perspectives. We also predict the population size of the elderly with different health statuses and their medical expenditure for the period 2020–2060. Feasible policy suggestions are provided in order to meet the challenge of rapid population aging.

The aging of populations brings a heavy burden to social old-age security and causes an increase in health care demands and the rapid expansion of medical expenses [3,4]. Scrutinizing the related literature, scholars have conducted empirical analysis on the health status of the elderly and discussed how the factors impact their health status. Scholars have also put forward policy implications related to medical care, pensions, and retirement to alleviate the burden of population aging [5,6,7]. Although the factors impacting health have been analyzed, a systematic theoretical framework is still lacking. The framework is the premise to analyze the relationship between elderly health and medical expenditure. Therefore, by referring to the medical behavior theory model of Anderson, this paper constructs a theoretical analysis framework of medical expenses for the elderly through propensity, enablement, and demand factors, which lay a solid foundation for the scientific prediction of the elderly population’s medical expenses.

According to the existing research, the factors impacting elderly health status can be roughly divided into demographic characteristics, socioeconomic status, lifestyle, and other factors. As to the demographic characteristics, the health condition of older men is significantly better than their counterparts [8,9]. In addition, the health status of the elderly generally decreases with age [10]. Even though the abilities of older people decline, they tend to report their health and life positively [11]. As to socioeconomic factors, the urban elderly are better off than those living in rural areas in terms of physical health, while this conclusion is reversed as to mental health [12]. Other social and economic factors, such as higher education and income, have a positive impact on elderly health [13]; however, Zachary Zimmer (2004) [14] found that the impact of these factors was not significant. In addition, support from family and community can help improve the health levels of the elderly [15,16,17]. As to lifestyle factors, a good lifestyle, such as taking part in physical exercises and entertainment, can help to improve the health levels of the elderly [18]. In addition, moderate alcohol drinking has little impact, but excessive alcohol drinking increases the morbidity and mortality of the elderly [19,20].

Accurate prediction of the health transition of the elderly is of great significance for analyzing their medical expenses. Through the literature review, it is found that scholars mainly make dynamic predictions for elderly health status from macro and micro perspectives. Due to the fact that the factors are multidimensional, the research methods adopted by scholars on this issue are very different. On the one hand, macro forecasting analysis selects key factors and sets key parameters to analyze the different situations of long-term care needs. Different studies referred to different bases when selecting key factors, and the key parameters were also set differently. For example, Comas Herrera (2007) [21] predicted the health care needs of the elderly in four European countries and set key parameters including lifestyle, nursing cost, health status, and external support. In contrast, Costa Font (2008) [22] used key parameters such as birth and death rates to simulate the population number and to calculate the population size of the elderly under different health conditions. On the other hand, micro prediction analysis is to build health status transition models and predict the health status of the elderly based on micro survey data. For example, Rickayzen et al. (2002) [23] first used the Markov method to predict the long-term care needs of the elderly. Peng Rong (2009) [24] adjusted the health transition probability of the American elderly population using the simple sequential method and predicted the long-term care needs of the Chinese elderly population. Chahed (2013) [25] and Hu (2015) [26] constructed logit regression and multiple regression models and made their predictions using the transition probability obtained from regression model simulation. Due to the complexity of the factors impacting health status, the factors are selected differently in different studies, thus the different results.

Based on the current research on the health and medical expenditure of the elderly, we propose the following summary: first, the health disparity of the elderly is studied as well as the factors impacting elderly health status, such as age, gender, urban or rural areas, and other socioeconomic factors. Second, scholars have suggested that the most important way to improve the health of the elderly is living a healthy lifestyle. Therefore, the popularization of basic medical and health education should be made, and an active and healthy lifestyle should be advocated. Third, scholars have used quantitative methods to analyze the long-term care needs of the elderly and predicted their health care demand and expenditure. Some scholars have predicted the health care needs of the elderly by setting key parameters and simulating the population size. It is generally recognized that the scientific assessment and prediction of the health care needed for the elderly is conducive, which provides the relevant information on the aging population in advance and gives data support for later policy interventions.

Although the current research on elderly health and its prediction has achieved some progress, many shortcomings remain, which are as follows: firstly, scholars have neither established a unified theoretical analysis framework to study the factors that impact elderly health nor incorporated the different health statuses of the elderly in the calculation of medical expenses. However, examining the health status of the elderly and its relation to medical expenditure is a key precondition to make an accurate prediction. Secondly, scholars have studied the health expenditure of the elderly, but an exact classification of health status has rarely been given. In addition, the healthy elderly population has generally not been included in the calculation. Although the per capita medical expenditure of the healthy elderly is lower, the total expenditure is much higher because of the large population, and its growth is fast, in line with the aging population. Thirdly, scholars have adopted the quantitative method in related research. However, the data sources and parameter settings are highly controversial, which may not reflect the actual social situation and may lack practical guiding significance.

This paper makes related improvements to the deficiencies mentioned above. First, we improve the elderly health transition matrix model and establish a theoretical analysis framework for elderly medical expenditure. Second, the health status of the elderly is classified using an authoritative database representing the actual situation of the elderly in China. Based on the scientific model and convincing data, the health transition probability and medical expenditure scale of the elderly in the period 2020–2060 are predicted. Third, according to the changing pattern of the elderly population in China, this paper predicts the changing trend of elderly population size and the medical expenses under different health conditions from 2020 to 2060. This paper also provides feasible policy suggestions on how to respond to the rising medical expenditure and the health care needed.

## 2. Data and Measures

### 2.1. Data

#### 2.1.1. Data Source

The data used in this article comes from the data project “China Health and Retirement Longitudinal Study (CHARLS)”. This project was jointly carried out by the Nation School of Development, Peking University, and the Institute of Social Science Survey, Peking University. This project aims to collect a set of high-quality microdata representing China’s middle-aged and elderly families, namely, individuals aged 45 and above, to analyze and promote research on population aging. CHARLS is one of the most authoritative databases on the aging problems of China, which is welcomed by academia domestically and abroad. The national baseline data of CHARLS was launched in 2011, and two follow-up surveys were conducted in 2013 and 2015. The surveys were conducted in 150 counties and 450 communities in 28 provinces or autonomous regions across China, covering approximately 19,000 respondents from 12,400 households. This article uses data from 2011–2015, taking from the sample the elderly people aged 60 and above as research objects. After eliminating the untraceable samples and any missing key information, we finally get 6812 valid cases.

#### 2.1.2. Ethical Statement

The authors of this manuscript predicted changes in medical expenditure by conducting a retrospective analysis of anonymous data obtained from a publicly accessible repository (i.e., CHARLS). Therefore, ethical clearance was waived by the institutional review board.

#### 2.1.3. Concept Definition

Regarding the definition of the health status of the elderly, this article refers to internationally accepted measurements based on the evaluation of the elderly’s activities of daily living (ADL) and instrumental activities of daily living (I-ADL). Alternatively, by observing whether the elderly need help in getting up, dressing, eating, bathing, going to the toilet, or doing indoor activities, housework, cooking, shopping, or taking medicine, the disability status of the elderly can be determined [3,4]. Due to the general damage process of elder health, I-ADL disorders come earlier than ADL disorders because one may not be able to cook (using cooking instruments), but he or she will still be able to eat (doing daily living activities). According to the ADL and I-ADL, the health status of the elderly can be determined, as shown in Table 1. According to the ADL and I-ADL conditions, the elderly’s health status can be divided into four categories: healthy (I), with mild disability (II), with moderate disability (III), and with severe disability (IV).

Regarding the definition of medical expenses for the elderly, we examine the actual medical expenses in the past year, mainly from the household and individual levels. As for the household level, the total direct expenditure of the elderly individual and the indirect medical expenditure of the family members in the past year in the CHARLS data are used as the main indicator. Direct medical expenses include medical expenses for outpatient visits, hospitalization fees, and daily drug purchases; indirect medical expenses include transportation expenses, nutrition expenses, and family care expenses. As to the individual level for the elderly, the total direct medical expenditure in the past year is used as an indicator, which includes direct expenses related to the medical expenses of the elderly, such as outpatient fees, hospitalization fees, and daily medical expenses. Other expenses such as caregiver salaries, transportation expenses for oneself or family members, and accommodation expenses are not included.

### 2.2. Improved Traditional Markov Model

Scholars often use the Markov chain to study the health transition probability from one state to another state. Based on this, utilizing Gagniuc’s model setting [27], we improved the traditional Markov chain and constructed the Markov model of the health state transition of the elderly. This model deduced the duration of different health states of the elderly and the number of elderly in different periods. Under the current condition of the health state of the elderly, the probability of the future health state can be predicted and analyzed. A stochastic process model is set up {X(t),t∈T}, where T = (0,1,2,3···), state-space I = (0,1,2,3···), if for any positive integer L, M, K and any $j_{l}>\cdots >j_{2}>j_{1}\left(m>j_{l}\right)$ and $i_{m+k},i_{m},i_{jl}\cdots ,i_{j2},i_{j1}$, then Equation (1) is established as below:

(1) $$\begin{array}{c} P\left\{X\left(m+k\right)=i_{m+k}|X\left(m\right)=i_{m},X\left(j_{l}\right)=i_{jl},\cdots ,X\left(j_{2}\right)=i_{j2},X\left(j_{1}\right)=S_{j1}\right\}\\ =P\left\{X\left(m+k\right)=i_{m+k}|X\left(m\right)=i_{m}\right\}\; \end{array}$$

It is a Markov chain when Equation (1) is established. The right end of Formula (1) means the probability P{X(m+k)=j|X(m)=i} when moving to j after k steps, namely, m + k, because of condition *i* at m step. The right end of the formula can be abbreviated as $p_{\;\mathrm{ij}}^{\left(k\right)}\left(m\right)$, which is the k-step transition probability of the Markov chain. Based on this, by improving the Markov chain and assuming K = 1, the health state transition matrix of the elderly is constructed as Equation (2):

(2) $$P=\left[\begin{array}{cccc} p_{11} & p_{12} & \cdots & p_{1n}\\ p_{21} & p_{22} & \cdots & p_{2n}\\ \vdots & \vdots & \ddots & \vdots \\ p_{n1} & p_{n2} & \cdots & p_{\mathrm{nn}} \end{array}\right]$$

Equation (2) is the transition matrix of the health status of the elderly, and P(m) = P^((1)) (m) is the transition matrix model.

### 2.3. TMP Model and Theoretical Framework

To calculate the medical expenditure for the elderly, we establish a systematic theoretical analysis framework. Whether medical expenses will be incurred is an optimal decision made by the elderly and their households. The acceptance decision of the medical treatments is impacted by factors such as income level, health awareness, and whether it is convenient to undergo medical care. Therefore, to avoid selective bias, the Two-Part model (TPM) is constructed for this analysis. Part 1: Whether medical expenses have occurred or not, which include the two conditions of “medical expenditure incurred” and “no medical expenditure incurred”. Part 2: The specific amount of medical expenditure if medical expenditure has been incurred.

In the first part of the TPM, the Probit model is used to investigate whether medical expenditure has occurred, as in Equation (3):

(3) $$I_{i}=x_{i}\delta_{1}+\mu_{1i}\;\mu_{1i}\sim N\left(0,1\right)$$

Among this, when I>0, it means that medical expenses have occurred.

The second part is a linear model of medical expenses, as in Equation (4):

(4) $$Y\left(ME_{i}|I_{i}>0\right)=x_{i}\delta_{2}+\mu_{2i},\;\mu_{2i}\sim N\left(0,\sigma_{\mu }^{2}\right)$$

In the process of empirical analysis, to systematically analyze the impact of the characteristics of the elderly on their medical expenditure, this paper establishes a theoretical analysis framework that includes predisposing, enabling, and demand factors. The details are shown in (5). The predisposing factors are related to the selective preferences of the elderly, including demographic and health behavior variables. The enabling factors are about affordability, mainly including variables related to socioeconomic status. Demand factors are mainly about the demand for medical services and mainly refer to health status variables. Based on these predisposing, enabling, and demand factors, a regression model is built, as in Equation (5):

(5) $$\;ME_{i}=f(H_{i},D_{i},HB_{i},SES_{i})$$

Among these, ME represents medical expenses; H represents the health status of the elderly; D is a set of demographic variables, including age, gender, and household registration; HB is a set of healthy behavior variables, including whether the elderly individual exercises, participates in social entertainment, or smokes; SES is a set of variables related to socioeconomic status, including education level, income level, and whether the elderly individual has pension and medical insurance.

## 3. Results

### 3.1. Analysis of the Elderly Health Status Transition and Medical Expenditure 

#### 3.1.1. Measurement of the Health Transition Rate of the Elderly Based on the Markov Model

The calculation of the health transition probability is the basis and prerequisite for analyzing the health transition of the elderly. CHARLS data were used to measure the health status of the elderly in 2011, 2013, and 2015, as shown in Table 2. In general, the proportion of the Chinese elderly population in a healthy (I) state remained steady—their percentage was 75% in 2011, and it decreased slightly to about 70%. Among the disabled population, compared with the higher proportion of mild disability (II), the proportions of moderate (III) and severe (IV) disability were much lower.

In fact, the aging process of the elderly is a dynamic evolution process, from a state of health (I) to mild disability (II), moderate disability (III), and severe disability (IV). According to the Markov transition matrix constructed in this paper, the disability rate of the elderly in 2011 is defined as vector A, and the disability rate in 2015 is defined as vector B, specifically as:

$$\begin{array}{c} A=(a_{1}\;a_{2}\;a_{3}\;a_{4})=\left(0.7505\;0.2052\;0.0317\;0.0126\right)\\ B=(b_{1}\;b_{2}\;b_{3}\;b_{4})=\left(0.7022\;0.2306\;0.0423\;0.0249\right) \end{array}$$

Therefore, bringing the A and B vectors into the Equation (2), the matrix P has the following form:

(6) $$P=\left[\begin{array}{cccc} p_{11} & p_{12} & p_{13} & p_{14}\\ p_{21} & p_{22} & p_{23} & p_{24}\\ p_{31} & p_{32} & p_{33} & p_{34}\\ p_{41} & p_{42} & p_{43} & p_{44} \end{array}\right]$$

In Equation (6), the sum of each row is equal to 1, satisfying $B=A*P^{4}$. At the same time, there is a certain transition probability between each group of P. The transition from the lighter disability state to the heavier disability state conforms to the law of descending order and vice versa. The optimal solution result of the transfer matrix is calculated by Matlab software, (MathWorks, Natick, Massachusetts, U.S.) as shown in Table 3. In order to verify the robustness of the *p*-value, we set the vector based on the elderly disability rate in 2013, and the settlement result is C=( 0.7107 0.2201 0.0393 0.0299 ); by comparing this with the actual data in 2013, the relative error is controlled within 5%, indicating that the measurement results are robust.

Table 3 reports the prediction results of the health transition probability of the elderly with different health statuses from 2011 to 2015. The results show that: first, among the transition of the elderly’s health status, each health status has the highest probability of maintaining its initial state. Take health (I) as an example. When elderly individuals are in a healthy (I) state, the probability of staying healthy (I) in the next period is as high as 82.15%, and the probability of transition to mild disability (II) is 14.87%. The probability of changing to moderate disability (III) is 2.64% and to severe disability (IV) is 0.34%. As time goes by, there is no rapid health deterioration of the elderly, although the deterioration is an inevitable one on the whole. It is realistic to maintain the health status of the majority of the elderly. The relevant departments of the government can make use of the healthy time of the majority of the elderly to formulate health policies, preparing for a future rapidly aging society.

Second, for the elderly with mild disability (II), the probability of health improvement that transforms to a healthy (I) state in the next stage is 27.11%. This figure is significantly higher than the probability of health deterioration to the moderate disability state (III) and severe disability state (IV), which are 1.97% and 0.47%, respectively. This fully demonstrates that the elderly, who account for more than 70% of the total disabled elderly, have a high possibility of improving their health through active daily care, health care, and exercise recovery. This group is the main target group for health improvement and the health policies of active intervention. In other words, the intervention policies applied to this group will be the most effective. Third, elderly people with severe disabilities have only a 1.31% chance to move back to being healthy (I). Alternatively, rehabilitation is rare for the severely disabled elderly. For this elderly group, it is essential to keep their health status stable, reducing their transfer probability to a more severely disabled state. At the same time, it is necessary to construct a specialized medical and health care system for the elderly with severe disabilities.

#### 3.1.2. Analysis of the Relationship between Health Status and Medical Expenses

Referring to the theoretical analysis framework constructed in this paper, the impact of different health conditions on family and individual medical behavior and medical expenses were examined based on the TPM, as shown in Table 4. Models (1) and (3) show the estimated results of the elderly who had treatments in the past year. The conclusions can be drawn as follows:

Firstly, as to the health status variables, the worse the health status of the elderly, the higher the probability that the family and the elderly had medical treatments, and the more the medical expenditure. Model 1 and Model 4 show that health status has a significant impact on family and individual acceptance of medical treatments and the higher expenditures. Specifically, with the health deterioration of the elderly, the probability of family medical participation increases 31.8%, and medical expenses increase by RMB 2792.1; at the individual level, these figures are 34.1% and RMB 4291.5. This fully shows that compared with other family members, the elderly accept more medical treatments as their health deteriorates. The key to improving the health status of the elderly is to provide accessible and affordable medical services. Therefore, improving the medical security system and medical service system for the elderly is an effective way to respond to their demand for health intervention.

Secondly, as to the demographic variables such as age, gender, and area, the findings are as follows: as age increases, the probability of family and individual medical participation has a downward trend. This is because deteriorating health conditions inevitably lead to increased healthcare costs, and a proportion of the elderly choose to restrain their medical treatments. Older women showed more active medical participation, and women were 0.061 times more likely to receive outpatient or hospital visits than older men. In addition, the medical expenses of the urban elderly were significantly higher than those of the rural elderly, and the expenditure of individual medical expenses was 2358.7 RMB/year higher. Rural elderly families showed a higher intensity of medical participation than their urban counterparts, but they spent less. Considering the socioeconomic divisions between the urban and rural elderly, it is concluded that rural families prefer to have outpatient or hospital care, but they have affordability problems. In addition, there are not as many good medical service provisions compared to urban areas. For the rural elderly, narrowing the income gap between urban and rural areas and ensuring adequate medical services are immensely significant to improving their health status.

Thirdly, as to socioeconomic status variables such as education, income, and insurance, the higher the education level, the higher the probability of medical treatments and medical expenses. In addition, there was a significant positive relationship between family income and the occurrence and expenditure of medical treatment. Furthermore, having a pension increased medical expenditure, which shows the income growth effect of pensions. Moreover, having medical insurance increases the probability of family and individual medical treatment, which reflects the health improvement effect of medical insurance. The findings demonstrate that establishing a social welfare system indeed helps improve the elderly participation rate in medical services and their health levels, but it also meant higher medical expenditure.

Fourthly, as to health-related behavior variables such as exercise, social activities, and smoking behavior, it was found that the elderly who exercised and participated in social activities showed more active medical participation. In addition, for the socially active elderly, their medical expenses were 1219.5 RMB/year higher than those who had infrequent social activities. In addition, elderly people who had smoking habits accepted more medical treatments, which is in line with the common sense that “smoking is harmful to health”. Unhealthy lifestyles damage the health status of the elderly and lead to an increase in medical services and expenditures. Therefore, advocating a healthy lifestyle can help improve individual health, which is also of great significance in alleviating medical service provision and expenditure.

### 3.2. Prediction of Health Status Transition and Medical Expenditure of the Elderly 

#### 3.2.1. Prediction of the Population Size of Different Elderly Health Statuses

According to the health transition results of the elderly in Table 2, the matrix P, which is the probability transition of elderly health status, is shown in Equation (7):

(7) $$P=\left[\begin{array}{cccc} 0.8215 & 0.1487 & 0.0264 & 0.0034\\ 0.2711 & 0.7045 & 0.0197 & 0.0047\\ 0.0991 & 0.1214 & 0.5936 & 0.1859\\ 0.0131 & 0.1599 & 0.3057 & 0.5213 \end{array}\right]$$

Each element in matrix P represents the probability of the health transition of the elderly from the base period to the current period, with the value 1 for each row in the matrix. According to this matrix’s results, the N-order transition probability of elderly health status can be simulated, i.e., the probability of the health transition of the elderly in different states in each T+4 year can be obtained. On this basis, according to the forecast data of the “Centennial forecast for the development trend of the aging population in China”, released by the China National Committee on Aging, the different health statuses of the elderly can be predicted in a queue based on the data in 2020 as the benchmark year. The distribution of the elderly population in different health states was simulated and predicted from 2020 to 2060, as shown in Table 5. 

Firstly, the total elderly population of China demonstrates an inverted “U” shape that will reach its peak around 2048, at about 434 million people. To be specific, China will witness its elderly population rising year by year from 2020 to 2040. The increase of 144 million of the elderly population in 20 years indicates how rapidly the population is aging in China. The government should pay full attention to population structure changes and the enormous impact on economic and social development. At the same time, policies to adjust the population structure should be issued, such as the policy to encourage childbirth. The social welfare system should be improved to alleviate the burden of pensions and medical care brought by the aging population. For the later 20 years, from 2040 to 2060, although the population size of the elderly population will decline, it remains above 400 million, which is still a large population. Policy intervention should improve the elderly care system and health care services to enhance their later life quality.

Secondly, as to the distribution of the elderly population in different health conditions, the proportion of the elderly in good health status is the highest, peaking at about 255 million, accounting for 59% of the total elderly population. Among the disabled elderly, the number of mildly disabled elderly is 121 million. The numbers for the moderately and severely disabled elderly are 37 million and 30 million, respectively. The number of mildly disabled elderly is much higher than that of moderately and severely disabled elderly. The health interventions to this group are the most effective, as mentioned. Therefore, active health care and health rehabilitation should be provided to effectively improve their health status.

Thirdly, in terms of elderly population growth trends between 2020 and 2060, the number of elderly with severe disability demonstrates a linear growth, increasing by 4.47 times over 40 years, exceeding the growth rate of the mildly and severely disabled elderly (1.89 and 3.28 times, respectively). This indicates that with population aging, there is an increase in life expectancy. However, the proportion of severely disabled elderly is not as large as the other counterparts. Its growth rate is the fastest. Their demand for medical services will increase correspondingly, which is also an important reason causing the growth of medical expenses. Therefore, on the one hand, it is vital to prevent the deterioration of the severely disabled status through active health prevention. On the other hand, a health service system that can provide scientific and professional long-term care needs to be established urgently.

#### 3.2.2. Prediction of Medical Expenditure for the Elderly in Different Health Conditions

Based on the health condition distribution of the elderly in the CHARLS data, the medical expenses for elderly families and individuals in 2015 were estimated, as shown in Table 6. With health deterioration, household and individual expenditures on medical treatments increased linearly. The household medical expenses of the elderly with severe disability were 1.94 times higher than those of healthy elderly. As to individual medical expenditure, the elderly with severe disability had expenditure 3.45 times higher than those who were healthy. It can be concluded that there are significant disparities in medical expenditure among the elderly groups of different health statuses. This conclusion is also proper for household medical expenditures. With the health deterioration of the elderly, solely relying on household affordability will not meet the needs for medical services. It is necessary to continuously improve the medical insurance system to cope with the risks of increasing medical expenses caused by health deterioration.

The measurement of medical expenditure should take into account the influence of price inflation and fluctuation. Therefore, the average annual economic growth rate from 2010 to 2020 is set at 6.5%. The annual rate for 2021–2030 is set at 5.4%, and for 2031–2040 and 2041–2060, the rates are set at 4.5% and 3.4%, respectively, because of the new normal economic development of China. In addition, taking into account the different medical services needed for the elderly in their different health conditions, the average annual medical expenses of the elder individuals when they are in J state during the period of T+4 are $ME_{j}\left(t+4\right)$. N(t+4) is the number of elderly people in each health state, and $\bar{ME}$ is the average annual medical expenses. The medical expenses of seniors aged 60 and above in China are recorded as Equation (8):

(8) $$ME_{j}\left(t+4\right)=\sum N\left(t+4\right)\cdot \bar{ME}$$

**Figure 1:**
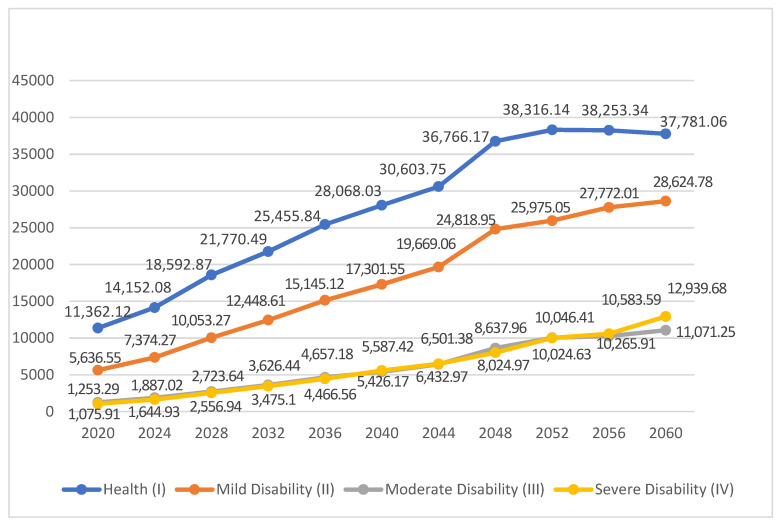
Prediction of annual medical expenditure for the elderly in China, 2020–2060.

According to the prediction results of the population size of the elderly in different health states, medical expenditure is calculated for 2020 to 2060, as shown in Figure 1. On the one hand, from 2020 to 2060, the average annual medical expenditure is highest for the elderly who are in healthy states, increasing from RMB 1.136212 to 3.778106 trillion. The large scale of medical expenditure for the healthy elderly is highly related to their much larger population. The medical expenditure of the elderly with mild, moderate, and severe disabilities increase to RMB 2.862478, 1.107125, and 1.293968 trillion, respectively, which increases 3.33, 8.83, and 12.02 times during the 40 years. Among the disabled elderly, the increased rate of medical expenditure of the severely disabled elderly is much higher, namely, 3.61 and 1.36 times that of the mildly and moderately disabled elderly. This fully shows that the worse the health status, the higher the medical expenses. Improving and keeping the health status of the elderly is an important measure to restrain the growth of medical expenditure.

On the other hand, as to the growth trend of medical expenses, with the overall expansion of the aging population, medical expenditure also expands correspondingly, especially for the healthy elderly and the mildly disabled elderly. Both increase significantly from 2020 to 2048, while later, after 2048, their growth rates slow down a bit. The average annual medical expenditure of the severely disabled elderly is not much different from that of the moderately disabled, but it will exceed the moderately disabled in 2056. At the late period of an aging society, attention should be paid to the high demand for health care for the severely disabled elderly because their demand for medical insurance will grow rapidly. The improvement of the medical insurance system and the establishment of a long-term care service system are required, especially, to meet their demand.

## 4. Conclusions and Policy Implications

Under the background of “Healthy China”, advanced age has become a new feature of population aging. With the increasing risk of the elderly’s health status deterioration, providing adequate health care services and effectively controlling the excessive growth of medical expenditure have become a top priority for China in the new era. This paper intends to estimate the size of the elderly population, their health status transition, and the medical expenditure involved. By improving the Markov matrix model and using the TPM, this paper constructs a theoretical analysis framework for the medical expenses of the elderly. This paper evaluates the size of the elderly population with different health states (the healthy, the mildly disabled, the moderate disabled, and the severely disable). It predicts the medical expenses for the period 2020–2060, following the data of CHARLS from 2011 to 2015.

The main findings of this research are as follows: (1) Under the aging trend. Although the elderly have a higher probability of maintaining their basic health status, their physical function decline is still inevitable. Especially for the severely disabled elderly, the rapid growth of population size and medical expenses will bring great pressure to the medical service system. (2) The health status of the elderly has a significant impact on their acceptance of medical diagnoses and treatments as well as medical expenditure. The worse the health status of the elderly, the more willing the families and individuals are to participate in diagnoses and treatments. This leads to higher medical expenditure that is increasingly beyond the affordability of individuals and families. (3) China’s elderly population shows an inverted “U” growth trend, reaching its peak around 2048, at about 434 million people. Among the elderly of different health statuses, the number of mildly disabled elderly will be 121 million. The number of moderately and severely disabled elderly will be 37 and 30 million, respectively. (4) In terms of medical expenditure, the total medical expenditure of the healthy elderly is the largest. This is because it is the largest population. As to the disabled elderly, the severely disabled elderly have the fastest increasing rate of medical expenditure.

To conclude, this study has important implications for making healthy aging policies and controlling the rapid growth of medical expenditure. Firstly, it is recommended to implement precise, classified, and targeted medical services for the elderly in their different health states. To the mildly disabled elderly, disease prevention and rehabilitation should come first because they still have higher probabilities of maintaining their health statuses and recovering healthy states. As to the moderately and severely disabled elderly, whose probabilities of health recovery are relatively small, it is necessary to provide daily care, medical care, spiritual consolations, and hospice care to improve the quality of their later lives. Secondly, there are significant disparities in medical participation and medical expenditure among the elderly living in urban or rural areas and those with different income levels. The medical and health care system should be reformed to improve the affordability of the elderly. The obstacles to the rural and low-income elderly population participating in medical care should be eliminated at the same time. Thirdly, the fast-rising medical expenditure should be shared among individuals, the government, and society. The medical insurance system should be reformed and improved; commercial medical insurance and social and medical treatment systems need to be developed. Fourthly, the elderly population size of China is expected to reach its peak in 2048, when it will face a high burden of pension and medical security. Therefore, strengthening the preventive interventions for the elderly and perfecting the medical service system before 2048 will help alleviate the challenges brought by ever-rising medical expenditure.

Last but not least, in order to scientifically deal with the health deterioration risks and medical expenditure expansion caused by the aging population, it is necessary to strengthen the basic population census and establish a dynamic monitoring mechanism for the disabled elderly. There is still a lack of data on the disabled elderly population. Conducting the census and statistics collections of the different populations can help us understand population distributions and trends, which are the premise for related policies. Therefore, subdivision statistics related to the elderly can be collected during the national census and published in the China population statistics yearbook. Subregional statistics or sampling statistics can also be collected. Furthermore, the elderly are encouraged to report their health status regularly, and a dynamic monitoring system for the disabled elderly should be established. In this way, the health status of the disabled elderly population can be estimated, and professional and targeted health care services can be provided.

## Figures and Tables

**Table 1:** Definition of the health status of the elderly.

Health Status	Description
Healthy (I)	Need no help in instrumental activities or daily living activities
Mild disability (II)	Need help in 1 or more instrumental activities, need no help in daily living activities
Moderate disability (III)	Need help in 1–3 daily living activities
Severe disability (IV)	Need help in 4 or more daily living activities

**Table 2:** Disability rate among the Chinese aged 60 and above, 2011–2015.

	2011	2013	2015
Healthy (I)	75.05%	72.31%	70.22%
Mild disability (II)	20.52%	21.73%	23.06%
Moderate disability (III)	3.17%	3.99%	4.23%
Severe disability (IV)	1.26%	1.97%	2.49%

Data source: CHARLS.

**Table 3:** Prediction of health status transition probability of the elderly.

Health Condition	Healthy (I)	Mild Disability (II)	Moderate Disability (III)	Severe Disability (IV)
Healthy (I)	0.8215	0.1487	0.0264	0.0034
Mild disability (II)	0.2711	0.7045	0.0197	0.0047
Moderate disability (III)	0.0991	0.1214	0.5936	0.1859
Severe disability (IV)	0.0131	0.1599	0.3057	0.5213

**Table 4:** Estimated relationship between health status and medical expenditure.

Independent Variable	Average Annual Medical Expenses for Families		Average Annual Medical Expenses for the Elderly	
	Participation Model (1)	Expenditure Model (2)	Participation Model (3)	Expenditure Model (4)
Health Status				
Status I–IV	0.318 *** (0.041)	2792.1 *** (207.8)	0.341 *** (0.027)	4291.5 *** (151.6)
**Demographic variables**				
Age 60 years old and above	−0.038 *** (0.017)	−183.2 * (146.8)	−0.035 *** (0.012)	−131.4 (146.5)
Gender 1 = Male; 0 = Female	0.057 (0.039)	−193.4 (155.6)	−0.061 * (0.028)	−255.3 (40.9)
Households Urban = 1; Rural = 0	−0.257 *** (0.053)	1135.0 *** (371.4)	0.132 *** (0.039)	2358.7 *** (90.4)
**Socioeconomic status variables.**				
Level of education 1 = Junior High School and above; 0 = other	0.021 (0.064)	218.7 (581.9)	0.052 * (0.037)	341.8 * (92.7)
Annual family income	0.057 *** (0.017)	2061.0 *** (143.5)	0.038 *** (0.013)	568.5 ** (37.2)
pension 1 = Yes; 0 = None	0.306 *** (0.068)	−2018.5 ** (611.7)	0.047 (0.045)	−2205.7 ** (105.6)
Health insurance 1 = Yes; 0 = None	0.475 *** (0.084)	994.9 (807.7)	0.232 * (0.078)	1385.5 * (257.4)
**Health behavior variables.**				
Physical exercise 1 = Yes; 0 = None	0.219 (0.114)	21.7 (24.4)	0.067 *** (0.048)	140.0 (80.3)
Social Activities 1 = Yes; 0 = None	0.072 (0.094)	−606.8 * (141.6)	0.261 *** (0.039)	−1219.5 * (71.2)
Smoking habits 1 = Yes; 0 = None	0.104 ** (0.087)	−226.3 (103.4)	0.072 * (0.049)	1291.3 (107.7)
Constant	−0.452 *** (0.305)	−7042.1 *** (1488.9)	−0.079 *** (0.262)	−7534.9 (430.5)
Sample value	1204	5608	1627	5185

Note: *, **, *** mean significance at the levels of 10%, 5%, and 1%, respectively; standard deviations are in parentheses.

**Table 5:** Population distribution prediction for the different health states, 2020–2060 (unit: 10,000 people).

Year	Healthy (I)	Mild Disability (II)	Moderate Disability (III)	Severe Disability (IV)	Total
2020	17,958.58	6253.92	1115.10	665.42	25,993.03
2024	19,053.55	6969.50	1430.15	866.59	28,319.79
2028	22,468.21	8528.18	1852.77	1209.07	34,058.22
2032	23,613.29	9478.42	2214.21	1474.91	36,780.83
2036	24,782.33	10,350.91	2552.28	1701.52	39,387.04
2040	24,964.58	10,802.49	2716.79	1944.61	40,428.47
2044	24,868.22	11,219.67	2942.60	2067.21	41,097.71
2048	25,486.19	12,077.20	3370.68	2446.67	43,380.73
2052	24,800.88	12,065.87	3487.22	2539.28	42,893.26
2056	23,586.41	12,020.60	3563.20	2553.49	41,723.71
2060	22,190.84	11,802.34	3660.55	2973.94	40,627.69

Note: Two decimal places are rounded off.

**Table 6:** Medical expenditure for seniors with different health states in 2015 (unit: yuan).

	Healthy (I)	Mild Disability (II)	Moderate Disability (III)	Severe Disability (IV)
Average annual family health expenditure	3754.79	4521.03	5699.14	7290.27
Annual individual medical expenditure	2572.06	4491.79	5540.11	8878.55

Source: Based on CHARLS data.

## Data Availability

Data available in a publicly accessible repository (China Health and Retirement Longitudinal Study (CHARLS)).
